# Inhibition of soluble TNFa in conjunction with anti‐amyloid immunotherapy

**DOI:** 10.1002/alz70855_107693

**Published:** 2025-12-25

**Authors:** Katelynn E. Krick, Joshua Lykins, Hsin‐Pei Wang, Erica M. Weekman, Donna M. Wilcock

**Affiliations:** ^1^ Indiana University School of Medicine, Stark Neurosciences Research Institute, Department of Anatomy, Cell Biology, and Physiology, Indianapolis, IN, USA; ^2^ Indiana University School of Medicine/Stark Neurosciences Research Institute, Indianapolis, IN, USA; ^3^ Indiana University, Indianapolis, IN, USA; ^4^ Indiana University School of Medicine, Stark Neurosciences Research Institute, Department of Neurology, Indianapolis, IN, USA; ^5^ Indiana University School of Medicine, Department of Neurology, Indianapolis, IN, USA

## Abstract

**Background:**

The approval of anti‐amyloid beta immunotherapies have showcased breakthroughs in our understanding and treatment of Alzheimer's disease (AD). While promising, anti‐amyloid beta (Aß) immunotherapies may lead to the development of ARIA (Amyloid Related Imaging Abnormalities). ARIA presents as cerebral edema (ARIA‐E) or hemorrhages (ARIA‐H), and no clear mechanism has been established. Neuroinflammation is a key etiology of AD that may contribute to the development of ARIA, particularly inflammation at the neurovascular unit. Thus, we hypothesize that inhibition of soluble TNFα (sTNFα) in combination with immunotherapy will reduce systemic inflammation and development of ARIA‐H.

**Method:**

In animal models, we can reliably recapitulate ARIA‐H with administration of 3D6, an Aß targeting antibody. Aged (19 mo) APP^SAA^ mice, a humanized Ab knock‐in model, received weekly injections of 3D6 (or IgG control) as well as a sTNFα inhibitor (or saline) over the course of 3 months. We used Congo red staining to quantify total, parenchymal, and vascular amyloid, flow cytometry to assess systemic inflammation via platelet activation, and Prussian blue to quantify microhemorrhage occurrence.

**Result:**

Administration of 3D6 reduced the average Congo positive vessel size as expected, however there were no significant differences in total Congo staining or percent of Congo at the vasculature between our groups. Administration of 3D6 increases superficial, cortical, and hippocampal bleeding compared to IgG controls following treatment from 19‐22 months of age. Interestingly, inhibition of sTNFα rescued 3D6‐driven hippocampal bleeds in these animals (*p* = 0.059). Additionally, platelets from 3D6 treated mice had higher surface levels of *p*‐selectin, a marker of activation, while sTNFα inhibition prevented this activation.

**Conclusion:**

Inhibition of sTNFα is promising as a potential combination therapy with anti‐Aß antibodies for reduction of systemic inflammation and microhemorrhage development, supporting future studies exploring this target.

